# First person – Julio Fierro Morales

**DOI:** 10.1242/bio.062275

**Published:** 2025-10-08

## Abstract

First Person is a series of interviews with the first authors of a selection of papers published in Biology Open, helping researchers promote themselves alongside their papers. Julio Fierro Morales is first author on ‘ Differential PaxillinB dynamics at *Dictyostelium* cell-substrate adhesions’, published in BiO. Julio conducted the research described in this article while a PhD student in Dr Minna Roh-Johnson's lab at the University of Utah, Salt Lake City, USA. He is now a postdoc in the lab of Dr Florentine Rutaganira at the Beckman Center, Stanford, USA, elucidating the evolution of molecular machinery such as cell-substrate adhesions using non-Metazoan model organisms.


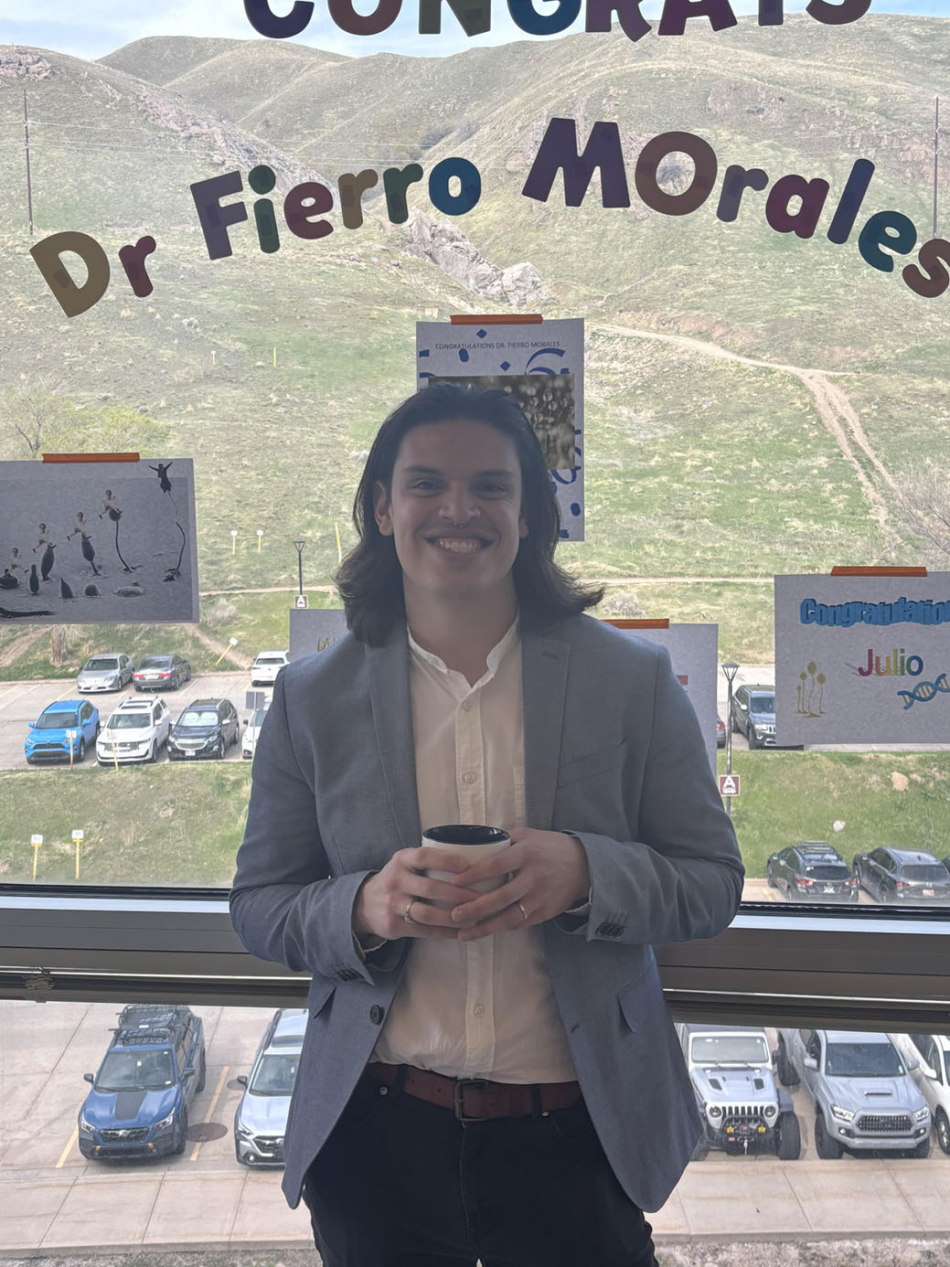


**Julio Fierro Morales**

**Describe your scientific journey and your current research focus**

I started my scientific journey as an undergraduate at Harvard, where I first dived into research investigating mechanisms of RNA in heritance in *C. elegans* under Dr Craig Hunter. This initial foray led me to want to gain more research experience and I joined Dr Marcus Pezzolesi's lab at the University of Utah to study the genetic aetiology of diabetic and non-diabetic kidney disease. After a couple of years as a lab tech in the Pezzolesi lab, I stayed at Utah to pursue my PhD. It was during my first year that I discovered my passion for cell biology thanks to a course taught in part by Dr Minna Roh-Johnson, whose lab I joined. After joining the Roh-Johnson lab, I combined my new love for cell biology with my ever-growing interest in evolutionary biology by pursuing an independent research project studying the evolution of focal adhesions for cell migration using the model Amoebozoan *Dictyostelium discoideum.* This work fostered a deep love for evolutionary cell biology, particularly using non-Metazoan organisms to elucidate how molecular toolkits have evolved for processes such as cell migration and development. After finishing my PhD this summer, I've now joined the FUNR lab at Stanford as a postdoc to study the evolution of cytoskeletal machinery in the choanoflagellate *Salpingoeca rosetta*, with a particular focus on elucidating the role this machinery plays in the transition of *S. rosetta* to a multicellular state.

**Who or what inspired you to become a scientist?**

I've always been fascinated by science and was privileged to have parents who helped foster this interest from a young age. One thing my parents always encouraged me to do is think outside the box; I feel that science is the perfect place to do that, and this mindset has motivated my use of non-Metazoan model organisms to elucidate how molecular mechanisms have evolved their functions and uses.

**How would you explain the main finding of your paper?**

Cells are capable of migrating in many different forms, one of which is called adhesion-based migration, in which cells adhere to the underlying substrate and generate forces to pull themselves forward, akin to a rock climber attempting to scale a boulder. Pivotal for this migration form are dynamic structures called cell-substrate adhesions, which are made of dozens of proteins and allow the cell to generate the forces necessary for migration. Though these adhesion structures and their properties have been extensively characterised and modelled in select lines of animal cell models, recent work suggests the composition and function of these adhesions during migration can drastically change depending on contexts such as cell type. One context where these cell-substrate adhesions are severely understudied is in non-animal cells. We previously characterised the composition of cell-substrate adhesions during cell migration in an amoeba called *D. discoideum*. We found reduced formation of cell-substrate adhesions of a specific composition – specifically the proteins PaxillinB and VinculinB – led to increased cell migration, a finding contrary to what has been reported in animal models. In this work, we delved deeper into the biology of *Dictyostelium* cell-substrate adhesions by asking how these different cell-substrate adhesion compositions impacted cell-substrate adhesion aspects such as their lifetime and the stability of their components at the structure. Interestingly, we observed that adhesions positive for PaxillinB but lacking VinculinB had a shorter lifetime than adhesions positive for both molecules, suggesting VinculinB plays a role in adhesion lifetime. Surprisingly, when we prevented VinculinB from localizing to adhesions by truncating the PaxillinB protein, we actually found the opposite finding, with these adhesions being longer lasting and PaxillinB being more stable. Though reconciling these findings can be somewhat unclear at first, we believe it's a clear indication that there are multiple proteins – including PaxillinB and VinculinB – involved in regulating *Dictyostelium* cell-substrate adhesions function, lifetime and stability during cell migration.

**What are the potential implications of this finding for your field of research?**

This finding suggests that the composition of *Dictyostelium* cell-substrate adhesions is a key factor in regulating adhesion dynamics and the stability of proteins at these adhesions during cell migration. Furthermore, it provides initial evidence of components such as VinculinB and PaxillinB being important factors in this regulation. We believe this initial finding is pivotal for understanding how *Dictyostelium* is able to regulate their adhesion structures in such a dynamic fashion and provide insight into how the regulation of these adhesions during cell migration has evolved. This in turn can be utilised to help guide research into cell-substrate adhesions in other largely under-investigated contexts such as leukocytes – which have historically been compared to *Dictyostelium* – or *in vivo*, where recent work characterizing cell-substrate adhesions in this context has shown some aspects of cell-substrate adhesions *in vivo* are actually somewhat akin to our previous findings in *Dictyostelium*.

**Figure:**
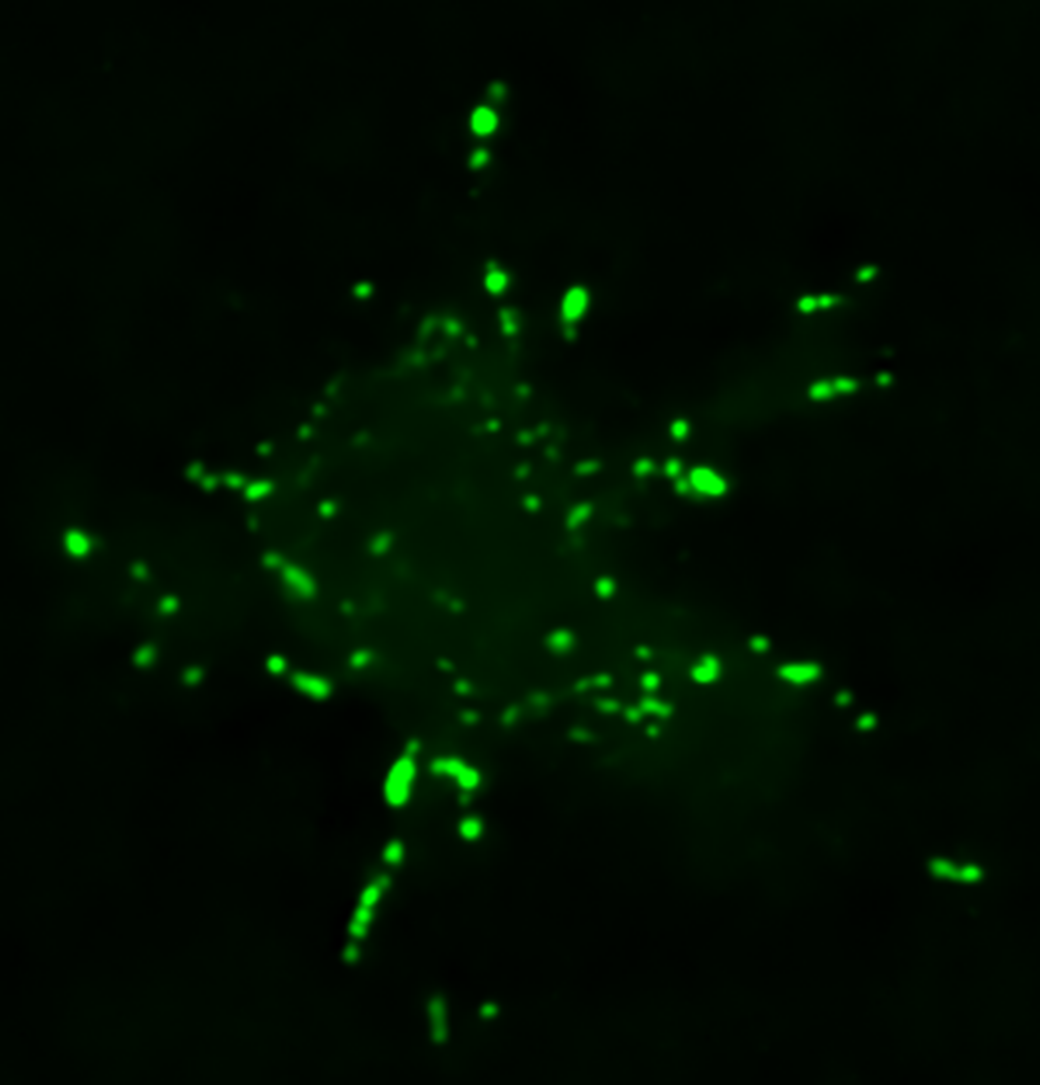
N-terminal truncation of GFP:PaxillinB leads to its hyper-localization to *Dictyostelium* cell-substrate adhesions, as evidenced by the large amount of GFP:PaxillinB punctae forming at the cell ventral surface.

**Which part of this research project was the most rewarding?**

Being able to learn new techniques such as FRAP and adapt them into model organisms where they are largely under-utilised such as *Dictyostelium*. I strongly believe non-Metazoan models are powerful organisms for studying the foundations of cell processes such as cell migration, cell adhesion, development, etc., and being able to utilise a variety of orthogonal techniques is something I believe will help develop the use of these organisms even further.

it's exciting to think that there is still so much out there that is yet to be discovered, and I get to contribute to it

**What do you enjoy most about being an early-career researcher?**

As daunting as it can feel sometimes, it's exciting to think that there is still so much out there that is yet to be discovered, and I get to contribute to it. I still get excited every time I find an interesting little titbit of data, or I observe something unexpected. As a trainee, I enjoy constantly expanding my network, hearing about novel research, and trying to figure out how I can incorporate it into my own research.

**What piece of advice would you give to the next generation of researchers?**

Research is a constant wave of ups and downs but if you keep at it and continue to pursue what's interesting to you, you'll hopefully find that both the ups and downs are a little bit higher than the previous one.

**What's next for you?**

I'm currently a new postdoc in the FUNR lab at Stanford's Department of Biochemistry, where I'm working on characterizing how cytoskeletal regulators and interactors in the choanoflagellate *S. rosetta* are important for cell processes such as transitioning to multicellularity, maintenance of a microvilli collar for capturing prey, and other outstanding questions. I hope to eventually become a PI and continue using non-Metazoan model organisms to elucidate the evolution of these molecular toolkits for cellular processes.
